# Development and implementation of “*Mazboot Bunyad*”: a digital health intervention for the screening and management of undernutrition among children aged 6–59 months, at private provider clinics in urban slums of Karachi, Pakistan

**DOI:** 10.1186/s44247-026-00303-7

**Published:** 2026-10-05

**Authors:** Shifa Salman Habib, Ammarah Ali, Muhammad Fazal Hussain Qureshi, Shehla Zaidi, Fasih Hansmukh, Wardah Ahmed, Tahira Hasham, Sajad Alam, Khalid Memon, Naveed Bhutto, Diwakar Mohan, Zahid Ali Memon

**Affiliations:** 1 Department of Community Health Sciences, Aga Khan University, Karachi, Pakistan; 2 University College London, Global Business School for Health, London, UK; 3 Department of International Health, Johns Hopkins Bloomberg School of Public Health, Baltimore, MD USA

**Keywords:** Undernutrition, Nutrition–immunization integration, Private sector engagement, Digital health intervention, Nutrition surveillance, Digital decision support system, Implementation research, Pakistan, Low- and middle-income countries, Nutrition registry

## Abstract

**Background:**

Undernutrition remains a leading driver of child mortality and morbidity in low- and middle-income countries, yet surveillance and case management in Pakistan still rely largely on infrequent surveys and paper-based registers that cannot track nutritional status in real time. The private sector, where most Pakistani households seek care, has been largely excluded from nutrition programming. We describe the development and early implementation of Mazboot Bunyad, a digital nutrition registry embedded within an integrated nutrition–immunization programme at private provider clinics in high-risk, low-income settlements of Karachi.

**Methods:**

The Android application and web dashboard used in this implementation study were designed through a participatory process using the AHEAD framework, in consultation with provincial health authorities, immunization staff, and community nutrition worker. The system used QR-linked child records, an embedded digital decision-support system that classified children by mid-upper arm circumference and has automated follow-up reminders. The tool was deployed across 33 clinics, and routinely collected programme data, a preliminary analysis of data from 1 July to 31 December 2024 were retrospectively reviewed.

**Results:**

During the period of 6 months, the *Mazboot Bunyad* application was used to screen and enroll 8,406 children aged 6–59 months. Field teams used the embedded Digital Decision Support System (DDSS) to classify children aged 6–59 months in real-time. Of these, 71.4% were classified as normal, 22.3% as moderately acutely malnourished (MAM), and 6.2% as severely acutely malnourished (SAM). The largest share of enrollments was among children aged 13–24 months (40.5%). Male children represented a slightly larger share of total enrollments (52.2%), but SAM burden was higher among females (7.3%) compared to males (5.3%). Supervisory teams used the dashboard to identify defaulters, monitor stock availability, and follow case recovery timelines.

**Conclusions:**

Early implementation of *Mazboot Bunyad* highlighted opportunities for enhanced nutritional surveillance using real-time tracking, but also challenges such as supply chain gaps that could affect the overall implementation. These offer valuable lessons for scaling digital tools in real world settings across Pakistan and other LMICs.

**Supplementary Information:**

The online version contains supplementary material available at https://doi.org/10.1186/s44247-026-00303-7.

## Background

Undernutrition is a significant public health challenge in low and middle income countries (LMICs), leading to higher mortality rates and long term morbidity, especially in children under 5 [1]. Pakistan is among the countries in the world with the highest rates of child undernutrition, and its progress remains slower than in other South Asian countries [1]. According to the National Nutrition Survey (NNS), 2018, 40.2% children under 5 years were stunted, while 17.7% had wasting [2]. In Sindh, the southern province of the country, the prevalence of undernutrition remains highest among all regions, with a striking 45.5% stunting and 23.3% wasting among young children. While the prevalence of undernutrition remains higher in rural settings of Pakistan, within urban areas it disproportionately affects children in low-income settings due to limited food security, lower buying power, and weak public health infrastructure [3]. In Karachi’s, urban low-income settlements, particularly those labelled as super high-risk and high-risk for polio, children face a dual burden of undernutrition and vaccine-preventable diseases [4, 5]. In these areas, fragmented service delivery and weak public primary health care systems, further exacerbate the challenges [6].

The urgency to develop sustainable, scalable strategies to address malnutrition is echoed in national and global policy discussions [7]. Sustainable Development Goals, such as SDG 2 (Zero Hunger) and SDG 3 (Good health and well-being) lay out the overarching goals addressing malnutrition [8]. Pakistan has developed policy and strategy documents to address malnutrition, including Pakistan’s Multi-sectoral Nutrition Strategy 2018–2025 [9] and Accelerated Action Plan for Reduction of Stunting and Malnutrition, Sindh [10]. Nutrition programming gained momentum after Pakistan became a signatory on Scaling-Up Nutrition (SUN) movement in 2013, however these remain targeted towards public facilities and are heavily donor dependent [11]. The unregulated private sector in Pakistan remains a blind spot for nutrition programming, despite approximately three-quarters of total health expenditure being incurred at private facilities [12].

Undernutrition is a complex and dynamic condition that requires continuous monitoring, timely interventions, and structured follow-ups to ensure effective management [13, 14]. Community based surveys, the last of which was conducted in 2018, remain infrequent and incapable of informing early intervention to address nutritional deficiencies in real time. Weak health information systems that depend on traditional paper-based recording mechanisms hinder the real time tracking of nutritional progress, monitoring treatment adherence, and assessing recovery outcomes [15]. Despite the success of electronic immunization registries [16], similar digital tracking mechanisms for nutrition interventions remain underdeveloped. Evidence from LMICs show that digital nutrition registries, when combined with strong implementation frameworks, can revolutionize malnutrition surveillance and case management [17].

### Digital registries for nutrition in LMICs

In recent times, LMICs including India [18], Bangladesh [19], Bhutan [20], Tanzania [21], Nigeria [22, 23] and Honduras [24] have launched digital nutrition registries with variable success. These registries vary considerably in design and functionality across key operational domains. India’s Poshan Tracker [18] and Bhutan’s growth monitoring platform [20] offer real-time, individual-level tracking; the former linked to unique national identification and generating alerts for missed visits and undernutrition risk, the latter flagging stunting through integration with the national health information management system. In contrast, Bangladesh’s Nutrition Information System dashboard [19], Nigeria’s DHIS2 nutrition module [22, 23], and Tanzania’s DHIS2-based system [21] primarily aggregate facility- and community-level indicators for periodic reporting, offering multi-level dashboards for managers and policymakers but limited patient-level follow-up. Honduras’ REDI-AN [24] demonstrates a cost-effective hybrid model, combining offline-capable mobile data entry by community health teams with integration into the national logistics system for supply management. Stock management remains the least consistently addressed domain, with only India, Honduras, and Pakistan’s Mazboot Bunyad tracking nutrition commodities at the facility or worker level. Mazboot Bunyad incorporates several features absent from most comparators QR code-based registration, defaulter tracking with alerts for severe and moderate acute malnutrition, geospatial identification of high-risk areas, and interoperable architecture while sharing the community health worker-centered access model common across these systems.

Pakistan’s District Health Information System 2 (DHIS2) [25] captures aggregate facility-level nutrition data through monthly reporting. It includes key indicators such as anthropometric measurements, deworming coverage, and Vitamin A supplementation. However, the system does not support patient-level tracking, real-time alerts, or integrated follow-up mechanisms. There is no embedded decision-support feature, and continuity of care is reliant on paper-based registers or Community Health Worker (CHW) recall. Supervisory dashboards are available but operate at a summary level. A real time digital registry would have the potential to address some the challenges with data systems in nutrition programs in Pakistan.

### *Mazboot Bunyad*: a digital health intervention for the screening and management of undernutrition in Karachi, Pakistan

The *Mazboot Bunyad* (Strong Foundation) digital nutrition registry was developed to equip community nutrition worker in the private sector, in low-income urban slums of Karachi with digital tools to identify and manage child undernutrition in line with WHO recommended standards. This digital intervention was embedded within an Integrated Nutrition-Immunization (INI) programme that engaged private sector providers in low-income settlements at high risk for polio, (High Risk and Super High-Risk Union Councils (UCs)), in Karachi. This paper is presented as a descriptive implementation research, and aims to document the design, operationalization, and early lessons from the implementation of *Mazboot Bunyad* intervention at 33 private provider clinics, located in low-income urban slums of Karachi, Pakistan.

## Methods

### Study setting and design

This descriptive implementation study with retrospective analysis of routinely collected program data was conducted in Karachi, the largest urban center of Pakistan, the fifth most populous country of the world [26]. Karachi, with a population of 20.3 million [27], is administratively spread over 7 districts and 178 UCs [28]. A UC is the basic structure of public administration and service delivery in Pakistan and a number of UCs combine to make a Taluka/Tehsil (sub-district). Of the forty Super High-Risk Union Councils (SHRUCs) that are under surveillance for Polio transmission in Pakistan, at least eight are in Karachi, where an additional thirty-four union councils have been declared High Risk Union Councils (HRUCs) [4]. Our intervention, *Mazboot Bunyad*, a digital health intervention targeting malnutrition, was rolled out in purposively selected 05 SHRUCS and 10 HRUCs. These UCs were selected in coordination with the Expanded Programme of Immunization (EPI), Sindh, based on their meagre performance in achieving EPI targets.

**Fig. 1:**
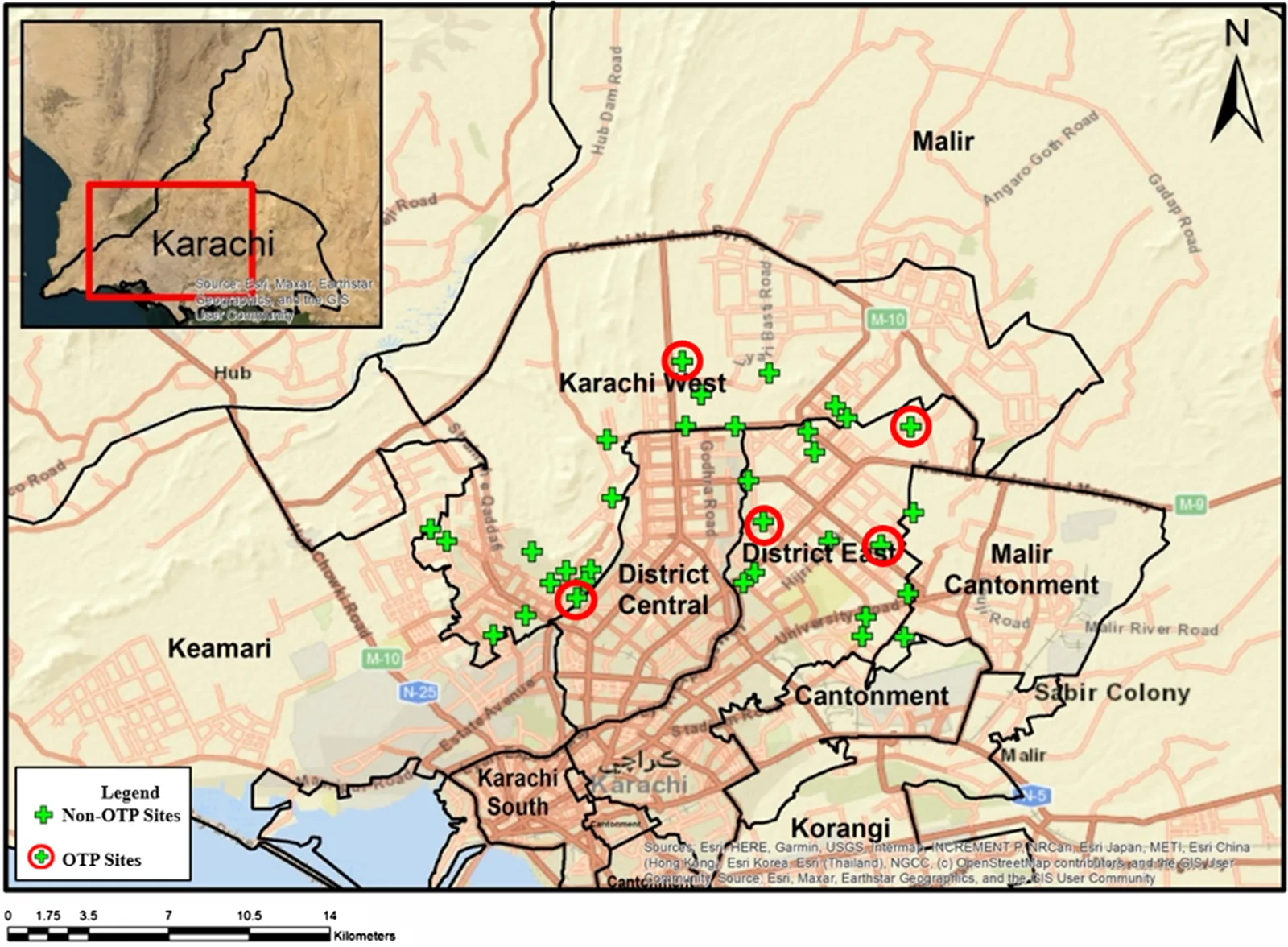
Geolocations of the 33 INICs located in Karachi, Pakistan

*Mazboot Bunyad* was embedded within a larger private provider engagement model, a collaboration between the Aga Khan University, Sindh Health Department and the Expanded Programme of Immunization (EPI), Sindh, that established 33 Integrated Nutrition Immunization centres (INICs) at existing private provider clinics in urban settlements of Karachi (Fig. 1), supplementing the existing curative services. Each clinic was refurbished with child friendly corners, deployment of additional human resource of 2 vaccinators and 1 female community nutrition worker for provision of integrated child health package that includes vaccination services, nutrition, infant young child feeding practices including counselling on breast feeding, and water, sanitation, and hygiene (WASH) counselling, at no service charge. Under the project, the EPI programme, Sindh is responsible for provision of vaccines, ice-lined refrigerators (ILRs), and other supplies. For the immunization component, the project uses the existing data management systems of the Government, whereas a new digital data management system for the nutrition component was established, and is described in this paper. In addition, the project employed an innovative social mobilization and digital communication approach for community awareness and engagement. Out of the 33 private provider clinics, 5 were designated as outpatient therapeutic programme (OTP) sites, where ready to use therapeutic food (RUTF) was made available, serving as referral sites for children with severe acute malnutrition (SAM). Data collected between the period of 01/07/2024 and 31/12/2024 was accessed for this research. This manuscript is reported in accordance with the mHealth Evidence Reporting and Assessment (mERA) checklist for mobile health interventions [29]. A completed checklist is provided as supplementary file.

### Mobile application design and development process

**Fig. 2:**
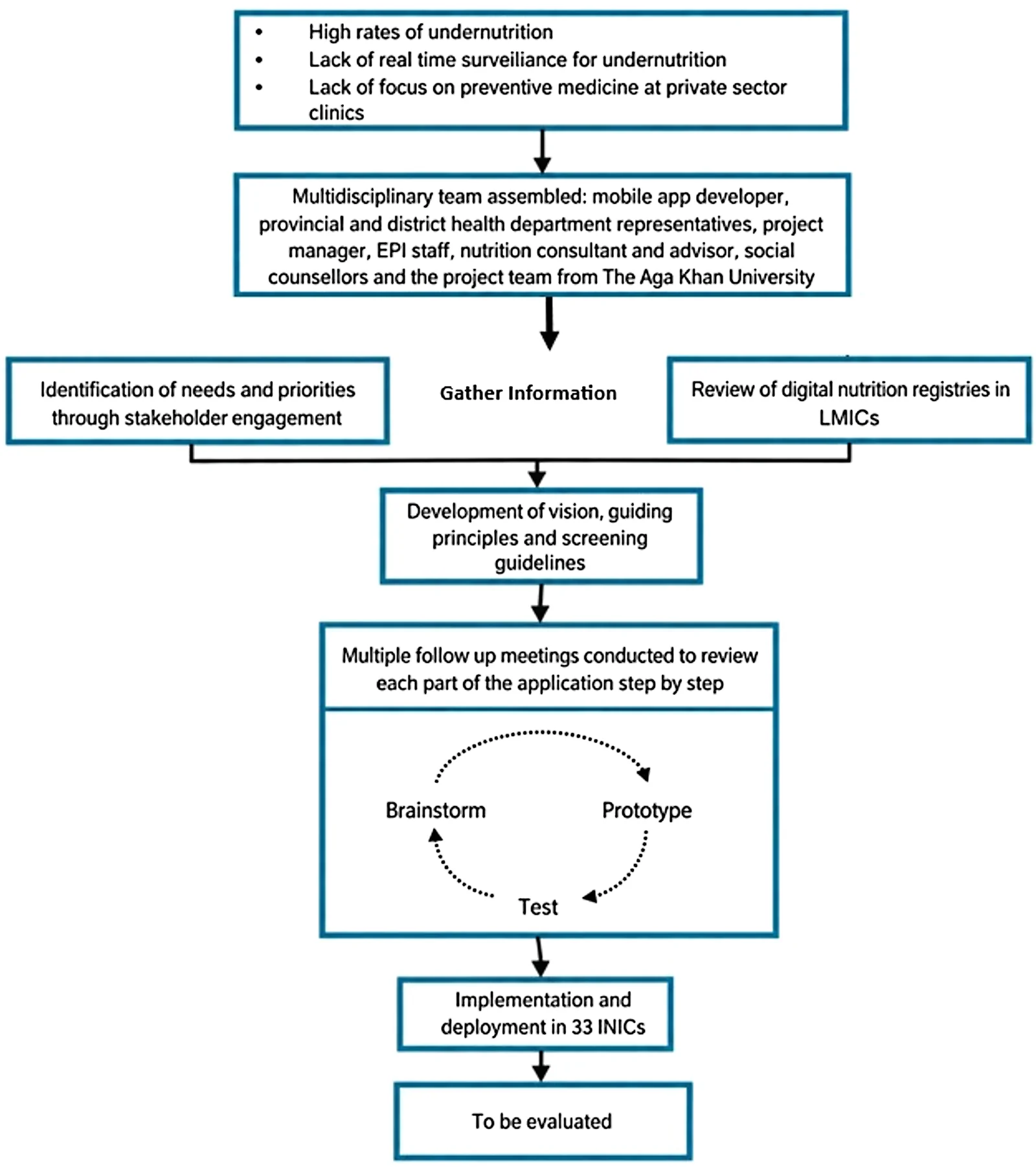
Steps in development of Mazboot Bunyad, adapted from the AHEAD framework [30]

The *Mazboot Bunyad* mobile application was designed through a participatory and user-informed process using the AHEAD framework [30], the steps of which are outlined in Fig. 2.

Firstly, we defined the problems to be tackled, that included high rates of undernutrition in children aged 6–59 months, lack of real time surveillance of nutritional status and lack of engagement of private providers in preventive health despite high utilization of private sector. Next, we assembled a multidisciplinary team that included a mobile application developer to lead the development process, representatives from the provincial and district health departments, EPI staff, nutrition consultant with 15 + years of experience, project managers and community nutrition worker and the project team from The Aga Khan University, that included health system experts. A nutrition advisor from the government was involved to help ensure that the screening algorithms aligned with national nutrition guidelines. Early in the development phase, consultative discussions were held with these stakeholders, to identify needs and priorities for the application’s function, which included offline data entry, automated anthropometric classification based on MUAC, and follow-up reminders. A review of digital nutrition registries of LMICs was done and Community based management of acute malnutrition (CMAM) guidelines were reviewed, while also conducting workflow observations at clinics and informal interviews with vaccinators, community nutrition worker, and managers to capture bottlenecks and user needs. Furthermore, during ideation and prototyping, the development team conducted multiple follow-up meetings to review each part of the application step by step, and feedback was collected both formally and informally to make improvements before deploying the application. Finally, the mobile application and web dashboard were rolled out across all 33 private provider clinics, where INIs were established. While evaluation was not the purpose of this paper, it was embedded into the study design and will be carried out in subsequent phases.

### Mobile application and systems overview

The *Mazboot Bunyad* mobile Android application was designed to capture data at each point of contact between community nutrition worker, caregivers, and their children.

**Fig. 3:**
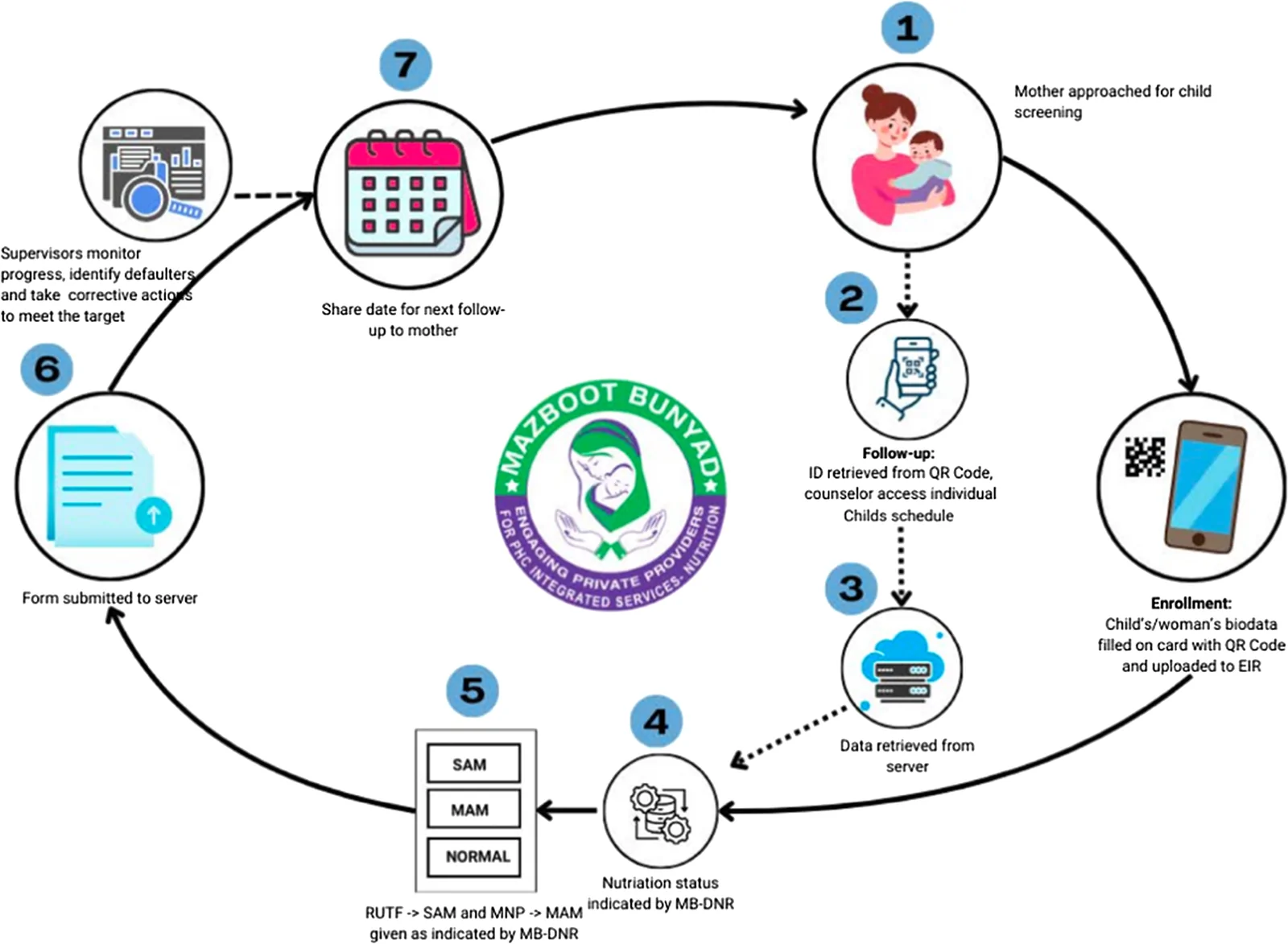
Overview of the Mazboot Bunyad system workflow

*Data flow* - At each visit by a new child, a unique QR code is generated and affixed to the child’s home-based record, linking the data to the electronic system, following informed consent obtained from parents or caregivers. During follow-up visits, the child’s record is retrieved by scanning the QR code, allowing the health worker to access the child’s nutrition history and recommended interventions. For each follow-up assessment new data are entered into the application and synchronized with the cloud server. All recorded data are consolidated on a web-based dashboard, that captures and displays comprehensive child details by fetching them through a QR code, which includes information such as name, gender, date of birth, snapshot, parent details, center information, and the date of entry, and enables instant access to their health records. This enables supervisors to monitor trends, track malnutrition cases, identify high-risk children, and take corrective action. Figure 3 summarizes the workflow under the *Mazboot Bunyad* intervention.

*Decision Support System* - The digital decision support system (DDSS), embedded within the app, utilizes built-in algorithms to classify children as SAM, moderate acute malnutrition (MAM) or healthy, using their mid-upper arm circumference (MUAC) measurements [31] and also generates the quantities of RUTF to be provided, based on this classification and the fed algorithms, in addition to providing customized nutrition counseling and referral recommendations. The tracking of nutritional improvement is displayed through a detailed timeline of each visit, showcasing the MUAC, weight, and height, along with any status changes from SAM to MAM to normal over time. The system automatically generates follow-up reminders for health workers update visit statuses along with tracking interventions. Additionally, it monitors stock levels of micro-nutrient powder (MNP) and RUTF per child visit and provides monthly overviews. The system logs each visit’s progress, showing how RUTF and MNP interventions contribute to a child’s recovery

*Monitoring* - To ensure proper monitoring and accountability, the system includes comprehensive reporting tools. There are monthly, weekly, and day-to-day reports available for community nutrition worker and supervisors to track child progress, monitor defaulters, and assess overall program impact. The dashboard also ensures efficient follow-up scheduling, and alerts for missed visits

### Screening procedures and algorithms

For nutrition screening, children aged 6–59 months were recruited from two main streams: those visiting the outpatient department (OPD) of private providers for curative services or accompanying other family members, and those presenting for routine immunizations at the INICs. Screening for malnutrition was conducted by trained community nutrition worker. Measurement of left MUAC was used as the measure of choicefor early detection of acute malnutrition [32] instead of Weight for Height Z score in accordance with CMAM guidelines, the national guidelines for screening and management of acute malnutrition, adopted by the Government of Pakistan.

At the time of first enrollment, every child was assigned a unique QR code and the child’s information, including age, weight, height, and MUAC were recorded digitally, while parents and caregivers were also issued a paper-based card for their records, that is updated on every fortnightly follow-up visit.

**Fig. 4:**
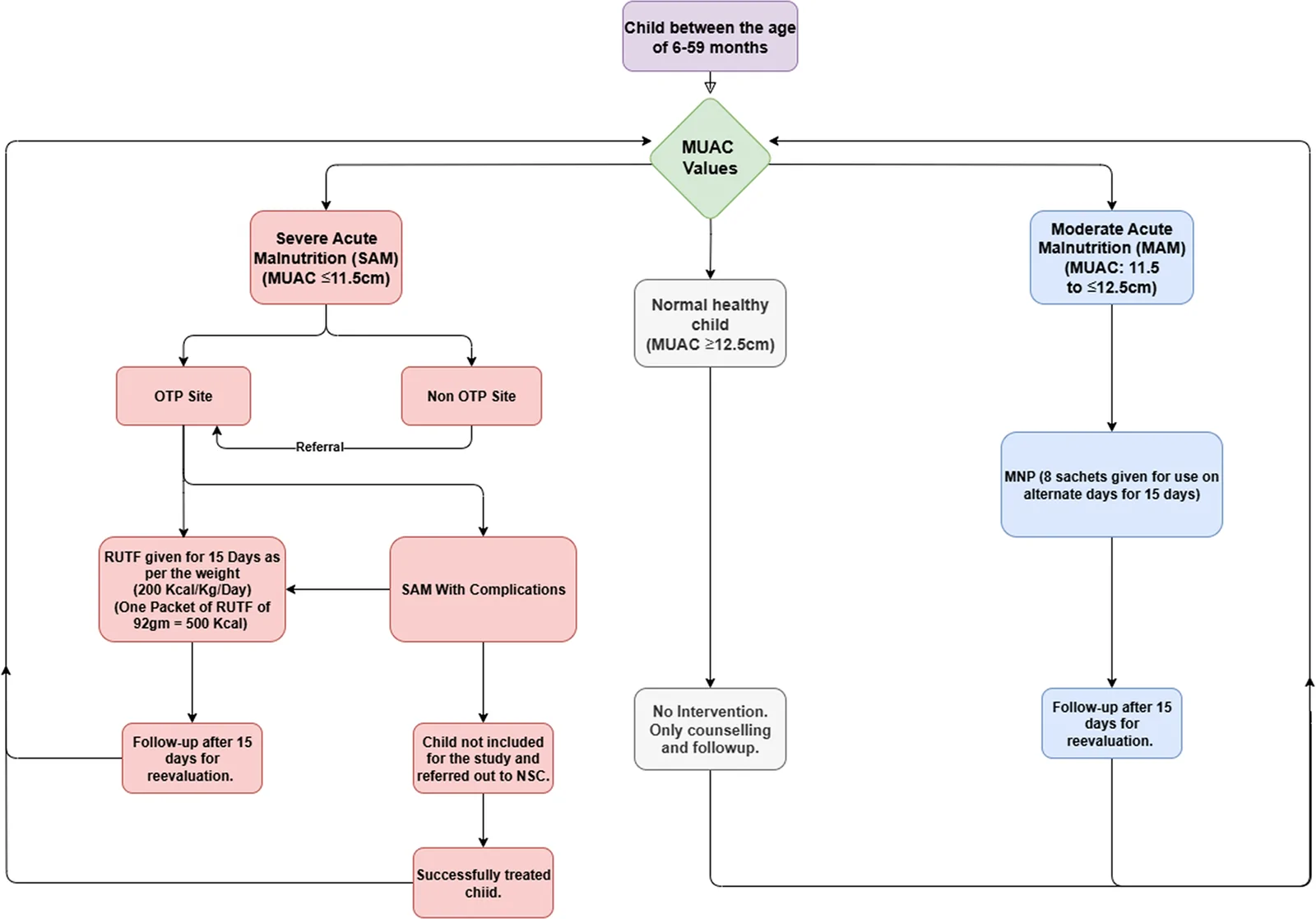
Screening and management algorithm followed at the INICs

Figure 4 below shows the screening and management algorithm for the intervention. Children, who visit the INICs, with a MUAC of 12.5 cm or above are considered well-nourished and receive dietary counseling and routine follow-up. Those identified with MAM (MUAC between 11.5 cm and 12.5 cm) are provided with MNP fortnightly for alternate days for 6 months, followed by reassessment. Children identified with SAM (MUAC less than 11.5 cm) are either managed at OTP sites with RUTF or referred to different Nutrition Stabilization Centers (NSC) established by the government, for specialized care, if complications are present. All children are re-evaluated after 15 days to monitor progress and determine further management. All families also receive counselling on the use of supplementary nutrition and the addition of essential nutrients in their diets, according to their specific context.

### Capacity building and training

As a critical component of the intervention rollout, a structured 5 days capacity building workshop was conducted for all frontline health workers, including the community nutrition worker responsible for child screening and caregiver counselling. These community nutrition workers received comprehensive training based on the CMAM guidelines [32], with the training content adapted in accordance with the workflows at the private provider clinics. The training was delivered by a certified nutrition consultant and emphasized early detection of malnutrition, classification of nutritional status, and appropriate counselling techniques. In addition to nutrition-specific instructions, community nutrition workers were educated on infant and young child feeding practices and essential WASH principles to promote holistic care for children under five. A dedicated training module also included hands-on training of the *Mazboot Bunyad* mobile application. This covered end-to-end use of the mobile application, including child registration, nutrition classification entry, follow-up tracking, and QR code generation. The practical sessions were conducted prior to the application’s official rollout, ensuring that community nutrition worker were equipped both with the clinical knowledge and digital proficiency required to deliver integrated nutrition services. After incorporating regular feedback from the community nutrition worker, refresher trainings were conducted periodically.

### Implementation strategy

Private provider clinics, previously selected as the Government of Sindh designated immunization clinics, served as the main sites for this intervention. Private providers serve as the primary point of care for many households in urban cities of Pakistan, where public sector infrastructure is scarce. The implementation of the original immunization programme began with mapping private clinics operating in SHRUCs and HRUCs that were eligible for registration with the Sindh Healthcare Commission (SHCC). Structured meetings and negotiations were held with the identified clinic owners to discuss service standards, reporting requirements and structured trainings leading to formal contracting-out agreements. Following contractual agreements, 33 private provider clinics in 05 SHRUCS and 10 HRUCs in the urban slums were transformed into INICs through collaborative partnerships with the Sindh Health Department and EPI Sindh. Refurbishment of the clinics including setting up of child friendly corners and hiring and capacity strengthening of additional human resources was done to enhance service delivery and community engagement.

## Results

### Demographic characteristics and nutritional classification

Data collected between the period of 01/07/2024 and 31/12/2024 was accessed, during which a total of 8,406 children under five years of age were enrolled in the *Mazboot Bunyad* digital registry. Of these, 4,386 (52.2%) were male and 4,020 (47.8%) were female. The largest proportion of enrolled children (40.5%) fell within the 13–24-month age group, followed by 25–36 months (28.0%) and 37–48 months (15.1%).

On initial MUAC-based screening, 6,003 children (71.4%) were classified as normal, 1,878 (22.3%) as having moderate acute malnutrition (MAM), and 525 (6.2%) as having severe acute malnutrition (SAM). Cases of both conditions were concentrated in the 13–24-month age group: of the 525 SAM cases, 59.2% (*n* = 311) fell in this band, as did 47.0% (*n* = 882) of the 1,878 MAM cases. The 49–59 month age group accounted for 1.7% of SAM cases and 4.4% of MAM cases. Examined as burden within each sex, girls carried a higher burden of both forms of acute malnutrition than boys: SAM affected 7.3% of enrolled girls (292/4,020) versus 5.3% of enrolled boys (233/4,386), and MAM affected 23.7% of girls (954/4,020) versus 21.1% of boys (924/4,386). Boys nonetheless made up a slightly larger share of total enrolment (52.2%, 4,386/8,406). Table 1 summarizes the demographic characteristics and nutritional classifications of the 8,406 children registered during the six-month period. These proportions describe the nutritional status of children presenting to the clinics through outpatient and immunization services and should not be interpreted as community-level prevalence. As a facility-based, help-seeking sample drawn from high-risk settlements, the cohort is likely to differ from the underlying population, and the figures represent the screened caseload rather than population burden.

**Table 1:** Sociodemographic characteristics of children aged 6–59 months registered in Mazboot Bunyad digital registry from 01 July 2024 to 31 December 2024

Variable	Nutritional Status						Total Cases (*n* = 8,406)	
	Normal (*n* = 6,003)		Moderate Acute Malnutrition (*n* = 1,878)		Severe Acute Malnutrition (*n* = 525)			
	*n*	%	*n*	%	*n*	%	*n*	%
**Gender**								
Male	3,229	53.8	924	49.2	233	44.4	4,386	52.2
Female	2,774	46.2	954	50.8	292	55.6	4,020	47.8
**Age Groups**								
6–12 months	30	0.5	11	0.6	4	0.8	45	0.5
13–24 months	2,209	36.8	882	47.0	311	59.2	3,402	40.5
25–36 months	1,541	25.7	640	34.1	173	33.0	2,354	28.0
37–48 months	1,015	16.9	229	12.2	24	4.6	1,268	15.1
49–59 months	815	13.6	83	4.4	9	1.7	907	10.8
Total	6,003	100	1,878	100	525	100	8,406	100

*percentages are rounded to one decimal place

**Fig. 5:**
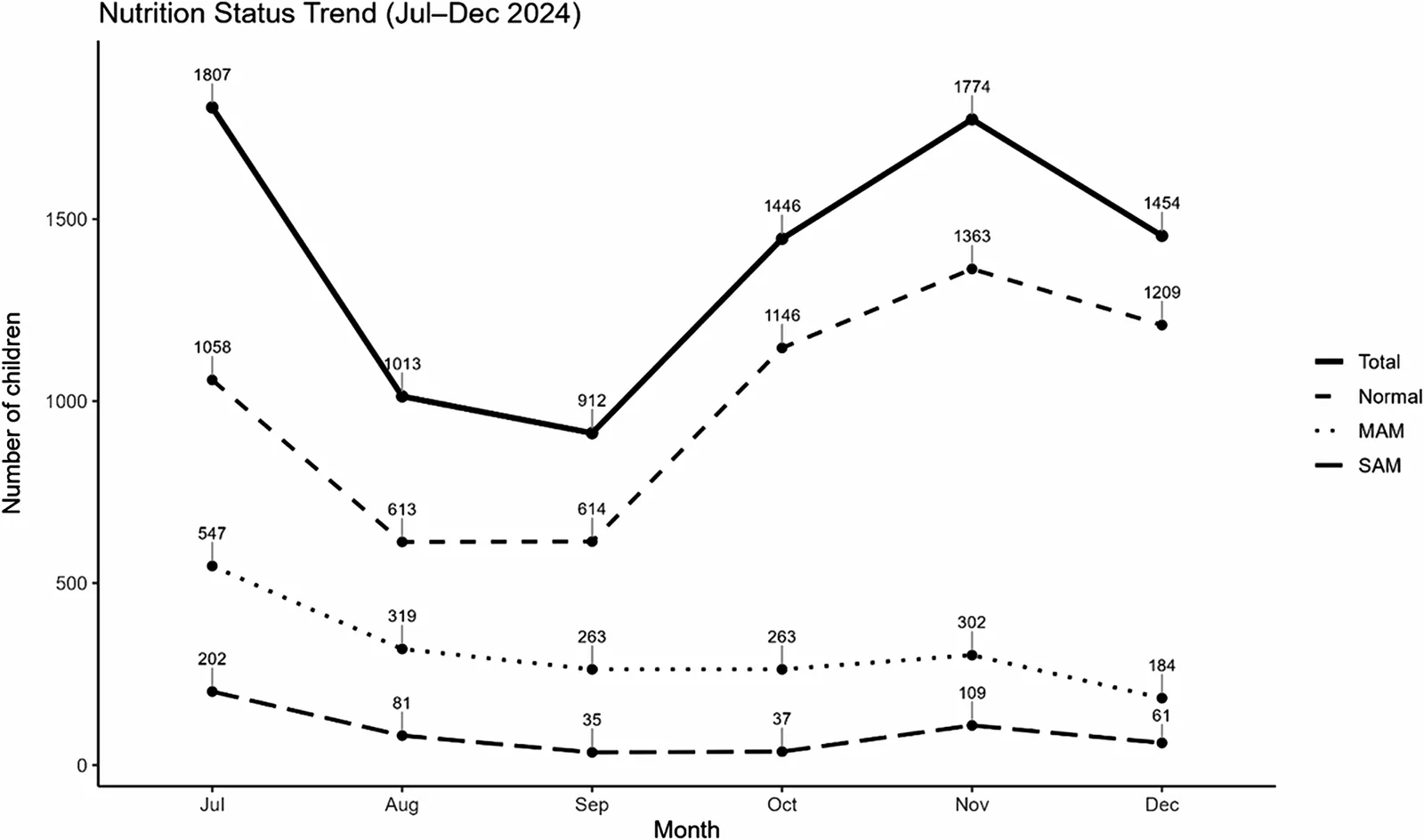
Time series showing number of SAM, MAM and normal children detected monthly from 01 July to 31 December 2024

As an additional descriptive analysis, Fig. 5 shows a time series description of the number of children aged 6–59 months undergoing nutritional screening and the screening results. A total of 1,807 children were screened in July, declining to 1,013 in August and reaching 912 in September. Numbers then rose to 1,446 in October, peaking at 1,774 in November, before slightly falling to 1,454 in December.

Across categories, children classified as normal decreased to 614 in September but increased steadily thereafter, reaching 1,363 in November. SAM cases also fell to their lowest in September (*n* = 35) before rising to 109 in November. Similarly, MAM cases declined to 263 in September and climbed to 302 in November. The dips in September coincided with temporary supply chain interruptions of RUTF. Enrollment and classifications subsequently recovered, and November reflected the highest overall screening volume and case detection across all categories.

## Discussion

Malnutrition remains a challenge of mammoth proportions in LMICs [33]. The implementation of the DHI *Mazboot Bunyad* demonstrates the potential and challenges of integrating low-cost digital solutions into routine child health services to improve the surveillance of malnutrition in LMIC settings. To our knowledge, digital health interventions for undernutrition that engage the private sector in Pakistan remain rare; this study contributes practical evidence on implementing such a tool within a private-provider network in high-risk urban settlements. Our study highlights the screened caseload of undernutrition in urban settings, where its often goes undetected without structured screening and individualized tracking and follow-up.

*The Mazboot Bunyad* intervention was embedded within the private healthcare sector that accounts for 62% of health care provision in Pakistan [34] and three quarters of health expenditure [12] but is often overlooked for the delivery of preventive health services. The differential incentives for the private health sector such as high patient volumes and unregulated revenues, have historically retained its focus on curative health services, which offer wider profit margins [35]. This project was one of its kind to integrate nutrition screening and management into a government-supported initiative in high-risk settlements, providing a unique model for public–private collaboration to address undernutrition. This is particularly important in the context of urban settings in Pakistan, where public primary health care infrastructure remains sparse [4]. This intervention, therefore, addresses a major blind spot in national nutrition programming. GAVI and other agencies have increasingly supported the approach of nutrition and immunization integration. Studies have shown that such integration improves coverage, streamlines delivery, and fosters more resilient health systems [36]. Our project also provided the opportunity for structured training, continuous monitoring and quality assurance at the private provider clinics, which often remain unregulated.

The importance of digitization in strengthening primary healthcare systems is increasingly well-established. For instance, in Ethiopia, mHealth interventions have shown promise in improving maternal and newborn health services, enhancing communication, reducing delays in care, and strengthening frontline decision-making in remote or underserved areas [37]. While not focused specifically on nutrition, such evidence supports the broader applicability of digital tools in community health interventions. In Pakistan, the *Zindagi Mehfooz* electronic immunization registry provides a successful example of how digital platforms can be leveraged for real-time child tracking, data-driven decisions, and service continuity [38]. Although ZM is immunization-focused, its QR-based architecture and scalable dashboard offer a valuable model for nutrition-related platforms like *Mazboot Bunyad*.

A core strength of the system is its DDSS, which enabled frontline providers to classify children using WHO growth standards and deliver individualized care plans with minimal delay. This real-time classification tool can potentially not only enhance diagnostic accuracy compared to paper-based methods, but also reduce the risk of treatment delays, by facilitating prompt referral of SAM cases. These findings align with emerging evidence from Pakistan, where DDSSs are being implemented and evaluated for usability, acceptability, and effectiveness in primary care contexts [39].

Additionally, the system’s cloud-based dashboard and stock management tools enabled supervisory teams to track service delivery, supply usage, and follow-up scheduling in real time. This type of analytics infrastructure is largely absent from traditional CMAM models in South Asia, which are still reliant on paper-based registers, fragmented reporting, and limited supervisory visibility [40].

Although this retrospective analysis does not yet include outcomes such as cure rates or recovery timelines, findings from similar interventions indicate likely benefits. For example, in Malawi, the use of RapidSMS (a mobile-based reminder system) significantly improved follow-up rates for malnourished children through automated alerts and tracking [41]. In India, the Dimagi-supported CommCare system demonstrated how mobile reminders and case tracking improved adherence to care and nutrition intervention timelines [42]. The Mazboot Bunyad platform incorporates the same mechanisms automated reminders and QR-linked patient records that drove these gains elsewhere, providing a plausible basis for similar benefits; however, follow-up and recovery outcomes were not measured in this implementation phase and are the subject of a planned evaluation.

### Limitations

*Mazboot Bunyad* was implemented as a service delivery enhancement rather than a randomized controlled trial hence, causal attribution of outcome changes solely to the DHI cannot be guaranteed. Additionally, while the digital tool improves classification accuracy and tracking, through embedded decision-support algorithms, further studies are needed to assess whether it also impacts the quality of anthropometric measurements, which remain human-dependent.

Our findings showed a decline in screenings in the initial phases of the programme, which is potentially linked with supply chain disruptions including shortages of RUTF. This shows how supplements such as RUTF may be used as a hook in the screening programmes as communities may have a preference for treatment over screening and counselling in isolation. Addressing such supply-side issues is essential, to ensure programme continuity. Findings from the Bangladesh NIS Dashboard reveal that digital nutrition platforms can be hampered by operational challenges even when technically robust [19].

During implementation, capacity gaps among community nutrition worker, particularly in obtaining accurate MUAC, weight, and height readings posed a significant challenge, which required active surveillance and corrective feedback mechanisms to be deployed for any quality assured dataset could be produced, To reduce this human dependency, future iterations could incorporate AI-assisted measurement tools, such as mobile-enabled height/weight capture systems or automated MUAC band readers.

Lastly, while the intervention demonstrated short-term functionality and acceptability within the private sector clinics, additional research is required to assess its long-term sustainability, cost-effectiveness, and integration into broader government sector MNCH programmes. These considerations are critical for informing future scale-up across diverse health system contexts. The screening was confined to children actively brought to participating clinics, our findings characterise the malnutrition caseload detected through facility-based screening, not the prevalence of acute malnutrition in these settlements. Help-seeking samples may over-represent unwell children while missing the most marginalised families who do not access care, so the proportions reported here should inform service planning rather than population-level estimates of burden. The study protocol was not prospectively registered. As a retrospective review of routinely collected programmatic data from a service-delivery intervention rather than a prospective trial, registration was not undertaken; this limits protections against selective reporting, and prospective registration is recommended for the planned evaluation phase.

## Conclusion

The experience of *Mazboot Bunyad* highlights how digital health tools, when embedded within existing care systems, can bridge critical gaps in child nutrition surveillance and strengthen frontline service delivery in underserved communities. However, these initiatives must be supported by sustained investments in supply chains and workforce capacity building. The model provides broader lessons for LMICs seeking to harness the potential of private sector actors for programmes of public health importance such as nutrition and immunization. Mazboot Bunyad was developed with the trust of the government and was operationalized through frontline private-sector workers; sustaining this partnership and acceptance across all stakeholders remains essential to demonstrate the tool’s impact on heath outcomes and wider scale-up.

## Supplementary Information

Below is the link to the electronic supplementary material.

Supplementary Material 1

## Data Availability

De-identified data underlying this article will be made available by the corresponding author upon reasonable request.

## Abbreviations

LMICs: Low- and Middle- Income Countries
CMAM: Community based Management of Acute Malnutrition
NNS: National nutrition survey
UC: Union council
SHRUC: Super high-risk union councils
HRUC: High-risk union councils
INIC: Integrated immunization nutrition centre
DDSS: Digital decision support system
SAM: Severe acute malnutrition
MAM: Moderate acute malnutrition
RUTF: Ready-to-Use Therapeutic Food
MNP: Micronutrient Powder
MUAC: Mid upper arm circumference
DHIS2: District health information system 2
SDG: Sustainable development goals
