# Supplementary material for: Development and implementation of “Mazboot Bunyad”: a digital health intervention for the screening and management of undernutrition among children aged 6–59 months, at private provider clinics in urban slums of Karachi, Pakistan
Source: BMC Digit Health. 2026 Oct 5;4(1):72. doi: 10.1186/s44247-026-00303-7 (PMC13638715; doi:10.1186/s44247-026-00303-7)
Supplement: Supplementary file 1 [file 44247_2026_303_MOESM1_ESM.pdf]

mHealth Evidence Reporting and Assessment (mERA) Checklist

Criteria	Item	mERA reporting requirement (condensed)	Page Number & location
**Infrastructure (population level)**	1	Availability of infrastructure (electricity, connectivity) in the study location to support technology operations.	Page 3-12<br>*Background; Study setting and design; Mobile application and systems overview*
**Technology platform**	2	Description and justification of software/hardware architecture, including any modifications to existing software.	Page 3-12<br>*Mobile application design and development; Mobile application and systems overview*
**Interoperability / HIS context**	3	Whether technical/structural integration into existing health information systems has been described, irrespective of whether achieved.	Page 3-6<br>*Digital registries for nutrition in LMICs (Table 1); Discussion*
**Intervention delivery**	4	Clear description of how the intervention is delivered: mode, frequency, timing, duration.	Page 9-11<br>*Screening procedures and algorithms; Mobile application and systems overview*
**Intervention content**	5	Details and source of the intervention content, and any modifications.	Page 9-11<br>*Screening procedures and algorithms; Capacity building and training*
**Usability / content testing**	6	Formative research and/or usability testing with the target group.	Page 8-9<br>*Mobile application design and development process*
**User feedback**	7	User feedback on, or satisfaction with, the intervention/interface.	Page 9-11<br>*Mobile application design and development; Capacity building and training*
**Access of individual participants**	8	Individual-level barriers/facilitators to adoption (affordability, social, structural).	Page 12-16<br>*Implementation strategy; Limitations; Discussion*
**Cost assessment**	9	Basic cost considerations of the intervention; reference to any formal economic evaluation.	**Not reported**<br>*Limitations (gap acknowledged)*
**Adoption inputs / programme entry**	10	How people are informed about the programme, including training and promotional activities.	Page 11-12<br>*Capacity building and training; Implementation strategy*
**Limitations for delivery at scale**	11	Clear presentation of the solution's limitations for delivery at scale.	Page 16<br>*Limitations*
**Contextual adaptability**	12	Adaptation (or not) of the solution to a different language, population or context.	Page 9-12<br>*Mobile application design and development; Capacity building and training*
**Replicability**	13	Detail (source code/screenshots/flowcharts/message examples) to support replication.	Page 9-11<br>*Figures 2-4; Mobile application and systems overview*
**Data security**	14	Description of data security and confidentiality procedures.	Page 9-11<br>*Ethical considerations; Statements and declarations*

Criteria	Item	mERA reporting requirement (condensed)	Page Number & location
**Compliance with national guidelines or regulatory statutes**	15	Mechanism ensuring content aligns with national/regulatory guidelines.	Page 11-12<br>*Screening procedures and algorithms; Capacity building; Implementation strategy*
**Fidelity of the intervention**	16	Whether the intervention was delivered as planned, and strategies used to assess fidelity.	Page 12-16<br>*Decision support / Monitoring; Limitations*

Source guideline: Agarwal S, et al. Guidelines for reporting of health interventions using mobile phones: mHealth Evidence Reporting and Assessment (mERA) checklist. BMJ 2016;352:i1174.
